# Autophagy in Pancreatic Cancer

**DOI:** 10.1155/2012/760498

**Published:** 2012-01-16

**Authors:** Daniel Grasso, Maria Noé Garcia, Juan L. Iovanna

**Affiliations:** Parc Scientifique et Technologique de Luminy, Stress Cellulaire, INSERM U624, 163 avenue de Luminy, CP 915, 13288 Marseille Cedex 9, France

## Abstract

Pancreatic adenocarcinoma (PDAC) is a devastating disease with an extremely poor life expectancy and no effective treatment. Autophagy is a process of degradation of cytoplasmic component capable of recycling cellular components or eliminate specific targets. The presence of autophagy in PDAC has been demonstrated. However, the implicated cellular pathways are not fully understood and, more importantly, the role of autophagy in PDAC is matter of intensive debate. This review summarizes recently published data in an attempt to clarify the importance of autophagy in this disease and try to reconcile apparently contradictory results.

## 1. Introduction

### 1.1. Autophagy

Macroautophagy (hereafter named autophagy) is a degradation process of cytoplasmic components, including entire organelles [1–3]. Autophagy starts with the presence of a single-membrane vesicle, the isolation membrane [2], which invaginates in order to sequester different targets into a double-membrane vesicle to form the autophagosome [4]. Eventually, autophagosomes fuse with lysosomes where the lysosomal hydrolases degrade the cargo [4]. Mechanistically, autophagy starts with the activation of ULK1 and ULK2 proteins which were kept inactive by mTOR activity [5, 6]. This event triggers the action of the ULK1/2-Atg13-FIP200-Atg101 complex that allows proper relocalization of a PI3KC3 (phosphatidyl-inositol-3-kinase—class III) from microtubules to endoplasmic reticulum (ER) to initiate vesicle nucleation [5–8]. The PI3KC3 complex also comprises p150, Ambra 1, and Beclin 1 proteins and generates phosphatidyl-inositol-3-phosphate in nucleation membrane to recruit additional autophagy-related (Atg) proteins to the site of autophagosome formation [9]. Afterwards, in an ubiquitination-like process, Atg12 is conjugated to Atg5, and the Atg12-Atg5 conjugated is associated with Atg16L1 which homodimerizes in a large structure named the Atg16 complex [10]. The Atg16 complex associates with the autophagosomal membrane where its activity is necessary for autophagosome membrane expansion and autophagy progression [10]. Moreover, in another ubiquitination-like process, LC3 protein is cleaved by Atg4 to expose a C-terminal glycine which is conjugated to phosphatidylethanolamine (PE) [11], allowing the recruitment of LC3-PE to autophagosome membrane. LC3-PE is considered as the most specific marker of autophagy [11]. Through this mechanism, autophagy was mostly considered as a mechanism allowing cells to recycle cellular component in order to generate energy during starvation conditions. However, the recent years have seen a revolution in autophagy with the demonstration that, in mammalian cells, it is a more complex and proactive system. In addition to its role during cell starvation, several reports evidenced a selective form of autophagy, capable of discriminating the target cargo for specific purposes or cellular requirements, with a clear implication in numerous human diseases [12–14]. For instance, selective autophagic degradation of mitochondria, called mitophagy, involves selective targeting and degradation of damaged mitochondria in Parkinson disease [15, 16]. All these exciting data about autophagy imply that it plays a role more important than expected in several human diseases, a very good reason for stepping up efforts to elucidate key autophagy mechanisms. Pancreatic cancer is not an exception, with numerous reports about autophagy associated with this devastating disease.

### 1.2. Pancreatic Adenocarcinoma

Although the incidence of pancreatic adenocarcinoma (PDAC) is the 10th among all cancers, it is the 4th leading cause of cancer deaths making PDAC a deadly disease with a relative 1-year survival rate of only 24% and an overall 5-year survival rate of 3 to 5%. It has a highly aggressive behavior with local invasion and distant metastases during the early stages of the disease [17, 18]. PDAC development is characterized by an almost constant sequence of gene mutations [19, 20]. Of these mutations, the first genetic alteration observed is a gain-of-function mutation of KRas which is present in nearly 100% in advanced PDACs. The mutated KRas protein constitutively triggers proliferation, differentiation, and survival signals. Hence, the KRas mutation is proposed as the initiating genetic lesion in PDAC. Moreover, homozygous deletion of the 9q21 locus is found in about 40% of tumors. Through different first exons and alternative reading frames, the 9q21 locus encodes the p16^INK4a^ and p19^ARF^ tumor suppressor proteins and therefore plays a key role in PDAC progression. Finally, p53 mutation and loss of SMAD4 are also frequently observed in the late stages of PDAC development [19, 20]. Morphologically, PDAC progresses from precursor lesions named “Pancreas Intraepithelial Neoplasias” (PanINs). PanINs show glandular pattern with duct-like structures and varying degrees of cellular atypia and differentiation [19, 20]. They are classified from Grade I, with presence of columnar mucinous epithelium to Grades II and III, with nuclear atypia. High grade PanINs transform into PDAC with areas of invasion beyond the basement membrane.

### 1.3. Autophagy in PDAC

The first indication of the presence of autophagy associated with pancreas malignancy was provided in 1999 by Réz and colleagues who showed images characteristic of autophagy in atypical acinar cell nodules [21]. Increased autophagic activity was observed by electron microscopy in premalignant cells, during progression of pancreatic adenocarcinoma induced in rats by azaserine and promoted by a row soya flour pancreatotrophic diet [21]. In premalignant cells, the total volume of autophagic vesicles increases upon treatment with vinblastine [21], a microtubule-disruptive drug commonly used to inhibit autophagosome-lysosome fusion [22]. Interesting details are provided by Fujii and colleagues who conducted a retrospective analysis of autophagy in human pancreatic tumor tissues [23]. They observed autophagy, characterized by LC3 immunohistochemistry, in patients before the beginning of their pharmacological treatment [23] and found a positive correlation between poor patient outcome and strong LC3 signal in the peripheral areas of pancreatic cancer [23] suggesting that the presence of autophagy in these areas could be associated with increased cancer progression.

## 2. Mechanisms and Molecules Involved in PDAC-Associated Autophagy

### 2.1. Hypoxia

PDAC is characterized by a very abundant stroma and poor vascularization. As consequence, PDAC cells are exposed to a shortage in nutrients and growth factors. Autophagy can be induced by hypoxia, which actually occurs if vascularization is inadequate. 

**Figure 1:**
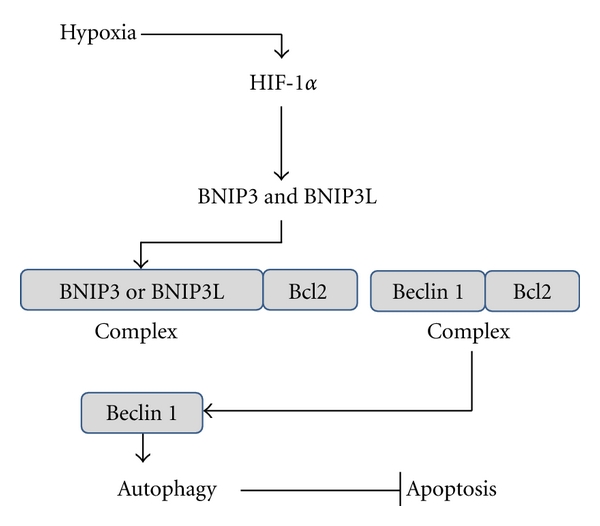
Hypoxia induces autophagy. Hypoxia activates HIF-1*α* which in turn induces expression of the BH3-containing proteins BNIP3 and BNIP3L to compete with Beclin 1 for its interaction with Bcl2. As a consequence Beclin 1 is released to induce autophagy.

 It seems to be dependent on the hypoxia-inducible factor-1 (HIF-1*α*) which is the master transcriptional regulator of the adaptive response to hypoxia [24]. Among target genes of the transcription factor HIF-1*α* are the genes encoding BNIP3 and BNIP3L, both proteins being required for hypoxia-induced autophagy [25]. Mechanistically, as Beclin 1, BNIP3, and BNIP3L possess a BH3 domain in their structure, and it is proposed that, through that domain, they compete with Beclin 1 for the interaction with Bcl2 and releasing Beclin 1, which induces autophagy [24, 26] (Figure 1). In agreement with this hypothesis, PDAC cells are characterized by high autophagic activity [27], a probable consequence of the hypoxic and starving conditions in which they are growing. Conversely, the behaviour of BNIP3 is characterized by a negative correlation between its expression and pancreatic cancer [28]. Interestedly, Okami and colleagues demonstrated that BNIP3 is silenced in PDAC by gene methylation, without downregulation of other HIF-1*α* target genes [28]. Moreover, specific BNIP3 downregulation is associated with gemcitabine resistance of pancreatic cancer cells [29]. In the work of Akada and colleagues comparing pancreatic cancer cell lines sensitive or resistant to gemcitabine, they identified by cDNA microarray the genes responsible for gemcitabine resistance [28]. They showed that BNIP3 expression was downregulated more than 90% in resistant cell lines and in those with intermediate sensitivity [28]. Nevertheless, microarray results indicated overexpression of BNIP3 in gemcitabine-sensitive pancreatic cancer cell lines [28]. Since BNIP3 is a hypoxia-inducible proapoptotic molecule, these results suggest that BNIP3 may have an important function during the initial stages of PDAC development, inducing autophagy and contributing to the response to hypoxia and starvation. As pancreatic cancer evolves, concomitant downregulation of BNIP3 makes it necessary that autophagy is induced by alternative pathways, as described hereunder.

### 2.2. Reactive Oxygen Species

**Figure 2:**
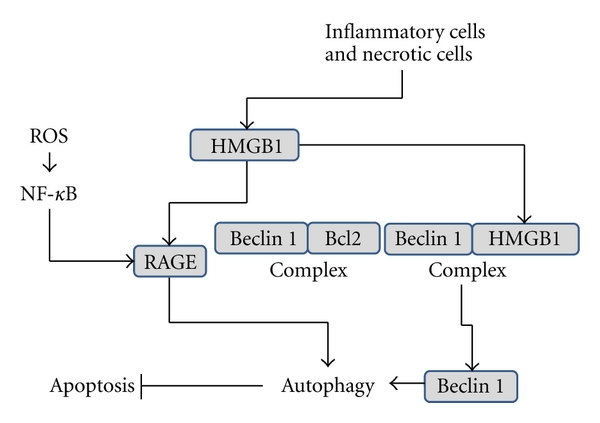
HMGB1 and RAGE induce autophagy. HMGB1 is released by necrotic and inflammatory cells. Expression of RAGE is induced by ROS in a NF-kB-dependent manner. On one hand, HMGB1 induces autophagy by its direct binding to the RAGE. On the other hand, HMGB1 induces autophagy by interacting with Beclin 1 which results in dissociation of Beclin 1-Bcl2 and subsequent induction of autophagy.

In recent years, reactive oxygen species (ROS) have gained an increased importance in tumor development. As demonstrated by DeNicola and colleagues, it is of vital importance for cancerous cells to keep under control their redox state [30]. They provide evidence that several oncogenes induce the antioxidant Nrf2 protein, in order to reduce ROS level [30]. Indeed KRas^G12D^/Nrf2^−/−^ mice present with a significant reduction in number of PanINs, highly supporting that ROS detoxification reduces tumorigenesis *in vivo* [30]. Moreover, the receptor for advanced glycation end products (RAGE) [31] and ROS could play a preponderant role in PDAC-associated autophagy. RAGE is a member of the immunoglobulin superfamily [32] implicated in ROS generation [33, 34] and in proinflammatory response [35, 36]. RAGE is overexpressed in PDAC, and it is associated with tumor resistance, proliferation, and invasiveness [37–40]. Furthermore, depletion of RAGE in PDAC cells increases sensitivity to chemotherapeutic agents [40], associated with caspase-3 cleavage [40]. On the contrary, overexpression of RAGE reduces apoptosis with a concomitant increase in autophagy [41]. Among ligands described for RAGE [32], the high-mobility group box 1 (HMGB1) plays a key role in PDAC. HMGB1 is a chromatin-associated nuclear protein involved in chromatin remodeling and regulation [42], which may also participate in inflammation and tumor progression [41, 42]. In fact, HMGB1 is released from necrotic and inflammatory cells, acting as an extracellular signaling molecule [41, 42]. HMGB1 has been proposed as mediator of pancreatic tumor cell resistance to antitumoral drugs [42] since interference RNA-mediated silencing of HMGB1 makes PDAC-derived cells more sensitive to the apoptotic cell death induced by melphalan treatment. The authors hypothesize that HMGB1 is released by necrotic tumor cells and enhances cell resistance by activating RAGE, inducing autophagy, and inhibiting apoptosis [40]. The molecular mechanism implicated in RAGE-HMGB1-mediated autophagy involves ROS. Kang and colleagues have demonstrated that PDAC cells exposed to H_2_O_2_ increase RAGE expression in a NF-kB-dependent manner [43]. Furthermore, PDAC cells show increased sensitivity to oxidative stress when RAGE is silenced [43]. In the same way, autophagy is induced in PDAC cells upon ascorbate treatment, but this effect is reversed by adenovirus-mediated downregulation of catalase expression [44, 45]. RAGE expression is upregulated by H_2_O_2_ treatment [43] through a pathway inhibited by inhibitors of the NF-kB pathway such as curcumin and Bay 11-7085 [46], or antioxidants such as N-acetylcysteine (NAC). Altogether, these results implicate the NF-kB pathway in RAGE-mediated autophagy and reveal a direct link between ROS and RAGE in PDAC. Kang and colleagues hypothesized that extracellular HMGB1, released by necrotic cells, is responsible for RAGE-mediated induction of autophagy in tumor cells [40]. However, they were not able to completely inhibit that effect with an anti-HMGB1 neutralizating antibody [40], suggesting the presence of at least one additional mechanism of action. In fact, Tang et al. gave evidence that endogenous HMGB1 may regulate autophagy by moving from nucleus to cytoplasm to interact with Beclin 1 in place of Bcl-2 [26, 47] (Figure 2). This is supported by the fact that HMGB1 translocation is induced by rapamycin and enhanced by ROS or by downregulation of superoxide dismutase [47]. Altogether, these data indicate that HMGB1 may play a double role in PDAC, on one hand by activating RAGE in neighbour cells and, on the other hand, by interacting with Beclin 1 in response to ROS.

## 3. Role of Autophagy in PDAC

**Figure 3:**
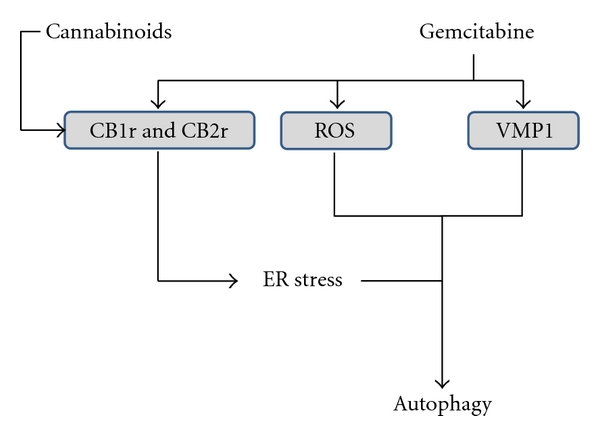
Chemotherapeutics agents induce autophagy. Gemcitabine induces expression of the autophagy starter protein VMP1 and CB1 and CB2 cannabinoids receptors, and production of ROS. Cannabinoids induce autophagy by activating ER stress and enhance gemcitabine action.

Although the role of ROS in autophagy induction is generally accepted, the role of autophagy in PDAC remains to be elucidated. Several lines of investigation are based on the ideas that autophagy is detrimental to tumor cells and that several antitumoral drugs act through this mechanism. In this way, it is important to note that chemotherapeutic agents generate ROS in patients [48]. Indeed, the effect of ascorbate on PDAC cells is totally dependent on H_2_O_2_ generation [44, 45]. Pardo and colleagues demonstrated in several PDAC-derived cell lines the induction of VMP1-mediated autophagy in response to gemcitabine treatment [49]. In this setting, gemcitabine-induced autophagy leads tumoral cells to apoptotic cell death. It is noteworthy that the inhibition of autophagy by 3-mehtyladenine or by knockingdown VMP1 reduces gemcitabine-induced apoptotic cell death [49]. These results are supported by data from Donadelli and colleagues who demonstrated an enhanced cytotoxic effect of gemcitabine when combined with cannabinoids, which induce ROS-mediated autophagy in pancreatic tumor cells [50]. Mechanistically, cannabinoid-dependent autophagy is induced by upregulating ER stress-associated genes such as p8, CHOP, TRB3, and ATF4 [51–53]. Interestingly, Donadelli showed that gemcitabine treatment activates expression of both cannabinoids receptors, CB1 and CB2, in a NF-kB-dependent manner [53]. In turn, cannabinoid treatment induces ROS production, ER stress, and autophagic cell death [50] (Figure 3). Again, this effect is inhibited when cells are treated with the free radical scavenger NAC [50]. Sulforaphane (SFN), a natural product extracted from broccoli, is able to eliminate highly resistant PDAC cells [54]. Naumann and colleagues showed that SFN induces autophagy and apoptosis in several PDAC-derived cells and, more interestingly, that autophagy and apoptosis, although independent from each other, are both dependent on ROS generation [55].

There is evidence suggesting that autophagy plays a role in PDAC cell survival in response to cell stress induced by ROS, tumor microenvironment, and antitumoral agents. For instance, the metastasis-suppressor KAI1 [56, 57] was shown to induce autophagy in PDAC cells, protecting them from apoptosis and growth inhibition [58]. The 2-deoxy-D-glucose, a glucose analog and glycolysis inhibitor, currently under clinical evaluation as chemotherapeutic drug, reduces cellular ATP and induces ER stress to eventually lead to cell death [59, 60]. In this context, cancer cells, including PDAC cells, respond to 2-deoxy-D-glucose by increasing autophagy in order to avoid ER stress, rather than compensating ATP depletion [60]. Moreover, Yang and colleagues show that autophagy is indeed requested for tumor development [27, 61]. They demonstrate that tumor cells derived from PDAC have a higher basal autophagy level than cell lines derived from other tumor tissues [27]. In fact, their results suggest that autophagy is not activated to control mitochondria homeostasis but to fuel oxidative phosphorylation [27]. However, when autophagy is blocked by chloroquine or by silencing ATG5, increased ROS detection is observed [27], indicating that autophagy may occur in response to oxidized reactive species as mentioned above. In addition, accumulation of autophagosomes and improvement of gemcitabine and 5-fluorouracile effect on pancreatic tumor cell lines were reported when they are combined with omeprazole [62]. Omeprazole is thought to interfere with lysosome homeostasis [63, 64] and ROS formation [64]. Altogether, these results support the hypothesis that autophagy is a survival reaction in response to ROS produced by antitumor drugs, leading to tumor cell resistance. 

Recently, Guo and colleagues have confirmed that Ras activation promotes cellular autophagy [65]. Working with epithelial kidney cells, these authors demonstrated that constitutively active Ras significantly increases basal autophagy with, however, concomitant limitation of starvation-induced autophagy. Importantly, depletion of Atg5 and Atg7, accompanied by accumulation of p62 and ubiquitinated aggregates, reduces tumoral growth [65]. Similar results are observed in p62^−/−^ cells [65]. These results indicate that the role of autophagy is not only to balance the higher metabolism of tumor cells but to buffer the higher energy demand by preserving the mitochondrial function [65]. Taking into account the role of ROS in PDAC, it is tempting to speculate about a yet unknown selective autophagy process able to eliminate ROS and other oxidized substrates.

**Figure 4:**
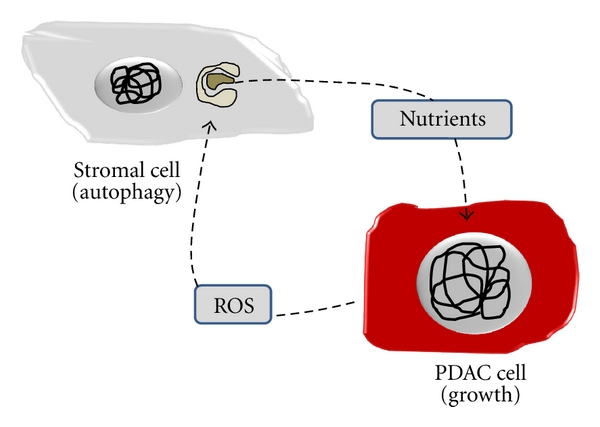
Autophagy of stromal cells fuel PDAC cells. PDAC release ROS to the tumoral microenvironment to induce autophagy in stromal cells. As consequence, stromal cells provide PDAC cancer cells with a steady stream of recycled nutrients and energy-rich metabolites.

Another point that needs to be considered is the role of the autophagy in PDAC stromal cells. Cancer cells activate autophagy in the tumor stromal compartment via paracrine mechanisms involving oxidative stress, as recently reviewed [66]. Autophagy in stromal cells provides PDAC cancer cells with a steady stream of recycled nutrients and energy-rich metabolites, which are reused by PDAC cells to drive tumor growth and metastasis (Figure 4). Thus, stromal catabolism fuels anabolic tumor growth. Therefore, inhibition of autophagy in the tumor stroma could stop or reverse tumor growth. This would explain the effectiveness of known autophagy inhibitors as antitumor agents, such as chloroquine and 3-methyladenine. Conversely, the induction of autophagy in epithelial cancer cells would block or inhibit tumor growth. This mechanism would explain the antitumor activity of agents that activate autophagy, such as mTOR inhibitors.

## 4. Conclusion

There is little doubt that autophagy plays a relevant role in PDAC development although several points remain to be clarified. Many efforts have been made in order to understand the mechanism(s) involved in the relationship between autophagy and PDAC, but elucidation is far from being completed. Data presented in this review let us to speculate on a bivalent participation of autophagy in PDAC cells. In this regard, autophagy may be a prosurvival process for tumor cells where it can fuel cell metabolism in the tumor microenvironment. However, autophagy can also be induced to reduce the oxidative stress generated by accelerated cell metabolism or chemotherapeutics treatments. On the other hand, the induction of autophagy in tumoral cells could lead to cell death. This model should resolve the “Autophagy Paradox,” where both inhibition and stimulation of autophagy have the same net effect, which is to inhibit tumor growth.

## References

[1] Farré JC, Krick R, Subramani S, Thumm M (2009). Turnover of organelles by autophagy in yeast. *Current Opinion in Cell Biology*.

[2] Yang Z, Klionsky DJ (2010). Mammalian autophagy: core molecular machinery and signaling regulation. *Current Opinion in Cell Biology*.

[3] Reggiori F, Klionsky DJ (2005). Autophagosomes: biogenesis from scratch?. *Current Opinion in Cell Biology*.

[4] Suzuki K, Ohsumi Y (2007). Molecular machinery of autophagosome formation in yeast, Saccharomyces cerevisiae. *FEBS Letters*.

[5] Hosokawa N, Hara T, Kaizuka T (2009). Nutrient-dependent mTORCl association with the ULK1-Atg13-FIP200 complex required for autophagy. *Molecular Biology of the Cell*.

[6] Ganley IG, Lam DH, Wang J, Ding X, Chen S, Jiang X (2009). ULK1.ATG13.FIP200 complex mediates mTOR signaling and is essential for autophagy. *Journal of Biological Chemistry*.

[7] Jung CH, Jun CB, Ro SH (2009). ULK-Atg13-FIP200 complexes mediate mTOR signaling to the autophagy machinery. *Molecular Biology of the Cell*.

[8] Mercer CA, Kaliappan A, Dennis PB (2009). A novel, human Atg13 binding protein, Atg101, interacts with ULK1 and is essential for macroautophagy. *Autophagy*.

[9] Wirawan E, Berghe TV, Lippens S, Agostinis P, Vandenabeele P (2012). Autophagy: for better or for worse. *Cell Research*.

[10] Mizushima N, Kuma A, Kobayashi Y (2003). Mouse Apg16L, a novel WD-repeat protein, targets to the autophagic isolation membrane with the Apg12-Apg5 conjugate. *Journal of Cell Science*.

[11] Kabeya Y, Mizushima N, Yamamoto A, Oshitani-Okamoto S, Ohsumi Y, Yoshimori T (2004). LC3, GABARAP and GATE16 localize to autophagosomal membrane depending on form-II formation. *Journal of Cell Science*.

[12] Vaccaro MI (2008). Autophagy and pancreas disease. *Pancreatology*.

[13] Cuervo AM (2004). Autophagy: in sickness and in health. *Trends in Cell Biology*.

[14] Levine B, Kroemer G (2008). Autophagy in the pathogenesis of disease. *Cell*.

[15] Youle RJ, Narendra DP (2011). Mechanisms of mitophagy. *Nature Reviews Molecular Cell Biology*.

[16] Vives-Bauza C, Przedborski S (2011). Mitophagy: the latest problem for Parkinson's disease. *Trends in Molecular Medicine*.

[17] Wray CJ, Ahmad SA, Matthews JB, Lowy AM (2005). Surgery for pancreatic cancer: recent controversies and current practice. *Gastroenterology*.

[18] Jemal A, Siegel R, Ward E, Hao Y, Xu J, Thun MJ (2009). Cancer statistics, 2009. *CA Cancer Journal for Clinicians*.

[19] Morris JP, Wang SC, Hebrok M (2010). KRAS, Hedgehog, Wnt and the twisted developmental biology of pancreatic ductal adenocarcinoma. *Nature Reviews Cancer*.

[20] Furukawa T, Sunamura M, Horii A (2006). Molecular mechanisms of pancreatic carcinogenesis. *Cancer Science*.

[21] Réz G, Tóth S, Pálfia Z (1999). Cellular autophagic capacity is highly increased in azaserine-induced premalignant atypical acinar nodule cells. *Carcinogenesis*.

[22] Köchl R, Hu XW, Chan EYW, Tooze SA (2006). Microtubules facilitate autophagosome formation and fusion of autophagosomes with endosomes. *Traffic*.

[23] Fujii S, Mitsunaga S, Yamazaki M (2008). Autophagy is activated in pancreatic cancer cells and correlates with poor patient outcome. *Cancer Science*.

[24] Mazure NM, Pouysségur J (2010). Hypoxia-induced autophagy: cell death or cell survival?. *Current Opinion in Cell Biology*.

[25] Tracy K, Dibling BC, Spike BT, Knabb JR, Schumacker P, Macleod KF (2007). BNIP3 is an RB/E2F target gene required for hypoxia-induced autophagy. *Molecular and Cellular Biology*.

[26] Pattingre S, Tassa A, Qu X (2005). Bcl-2 antiapoptotic proteins inhibit Beclin 1-dependent autophagy. *Cell*.

[27] Yang S, Wang X, Contino G (2011). Pancreatic cancers require autophagy for tumor growth. *Genes and Development*.

[28] Okami J, Simeone DM, Logsdon CD (2004). Silencing of the hypoxia-inducible cell death protein BNIP3 in pancreatic cancer. *Cancer Research*.

[29] Akada M, Crnogorac-Jurcevic T, Lattimore S (2005). Intrinsic chemoresistance to gemcitabine is associated with decreased expression of BNIP3 in pancreatic cancer. *Clinical Cancer Research*.

[30] DeNicola GM, Karreth FA, Humpton TJ (2011). Oncogene-induced Nrf2 transcription promotes ROS detoxification and tumorigenesis. *Nature*.

[31] Neeper M, Schmidt AM, Brett J (1992). Cloning and expression of a cell surface receptor for advanced glycosylation end products of proteins. *Journal of Biological Chemistry*.

[32] Bucciarelli LG, Wendt T, Rong L (2002). RAGE is a multiligand receptor of the immunoglobulin superfamily: implications for homeostasis and chronic disease. *Cellular and Molecular Life Sciences*.

[33] Basta G, Lazzerini G, Del Turco S, Ratto GM, Schmidt AM, De Caterina R (2005). At least 2 distinct pathways generating reactive oxygen species mediate vascular cell adhesion molecule-1 induction by advanced glycation end products. *Arteriosclerosis, Thrombosis, and Vascular Biology*.

[34] Cai W, He JC, Zhu L, Lu C, Vlassara H (2006). Advanced glycation end product (AGE) receptor 1 suppresses cell oxidant stress and activation signaling via EGF receptor. *Proceedings of the National Academy of Sciences of the United States of America*.

[35] Alexiou P, Chatzopoulou M, Pegklidou K, Demopoulos VJ (2010). RAGE: a multi-ligand receptor unveiling novel insights in health and disease. *Current Medicinal Chemistry*.

[36] Rojas A, Figueroa H, Morales E (2010). Fueling inflammation at tumor microenvironment: the role of multiligand/rage axis. *Carcinogenesis*.

[37] Logsdon CD, Fuentes MK, Huang EH, Arumugam T (2007). RAGE and RAGE ligands in cancer. *Current Molecular Medicine*.

[38] Abe R, Yamagishi S (2008). AGE-RAGE system and carcinogenesis. *Current Pharmaceutical Design*.

[39] Fuentes MK, Nigavekar SS, Arumugam T (2007). RAGE activation by S100P in colon cancer stimulates growth, migration, and cell signaling pathways. *Diseases of the Colon and Rectum*.

[40] Kang R, Tang D, Schapiro NE (2010). The receptor for advanced glycation end products (RAGE) sustains autophagy and limits apoptosis, promoting pancreatic tumor cell survival. *Cell Death and Differentiation*.

[41] Tang D, Kang R, Zeh HJ, Lotze MT (2010). High-mobility group box 1 and cancer. *Biochimica et Biophysica Acta*.

[42] Liu Y, Prasad R, Wilson SH (2010). HMGB1: roles in base excision repair and related function. *Biochimica et Biophysica Acta*.

[43] Kang R, Tang D, Livesey KM, Schapiro NE, Lotze MT, Zeh HJ (2011). The receptor for advanced glycation end-products (RAGE) protects pancreatic tumor cells against oxidative injury. *Antioxidants and Redox Signaling*.

[44] Du J, Martin SM, Levine M (2010). Mechanisms of ascorbate-induced cytotoxicity in pancreatic cancer. *Clinical Cancer Research*.

[45] Cullen JJ (2010). Ascorbate induces autophagy in pancreatic cancer. *Autophagy*.

[46] Pahl HL (1999). Activators and target genes of Rel/NF-*κ*B transcription factors. *Oncogene*.

[47] Tang D, Kang R, Livesey KM (2010). Endogenous HMGB1 regulates autophagy. *Journal of Cell Biology*.

[48] Ozben T (2007). Oxidative stress and apoptosis: impact on cancer therapy. *Journal of Pharmaceutical Sciences*.

[49] Pardo R, Lo Ré A, Archange C (2010). Gemcitabine induces the VMP1-mediated autophagy pathway to promote apoptotic death in human pancreatic cancer cells. *Pancreatology*.

[50] Donadelli M, Dando I, Zaniboni T (2011). Gemcitabine/cannabinoid combination triggers autophagy in pancreatic cancer cells through a ROS-mediated mechanism. *Cell Death and Disease*.

[51] Carracedo A, Gironella M, Lorente M (2006). Cannabinoids induce apoptosis of pancreatic tumor cells via endoplasmic reticulum stress-related genes. *Cancer Research*.

[52] Carracedo A, Lorente M, Egia A (2006). The stress-regulated protein p8 mediates cannabinoid-induced apoptosis of tumor cells. *Cancer Cell*.

[53] Salazar M, Carracedo A, Salanueva J (2009). Cannabinoid action induces autophagy-mediated cell death through stimulation of ER stress in human glioma cells. *Journal of Clinical Investigation*.

[54] Kim MK, Park JH (2009). Conference on "Multidisciplinary approaches to nutritional problems". Symposium on "Nutrition and health". Cruciferous vegetable intake and the risk of human cancer: epidemiological evidence. *Proceedings of the Nutrition Society*.

[55] Naumann P, Fortunato F, Zentgraf H, Büchler MW, Herr I, Werner J (2011). Autophagy and cell death signaling following dietary sulforaphane act independently of each other and require oxidative stress in pancreatic cancer. *International Journal of Oncology*.

[56] Liu WM, Zhang XA (2006). KAI1/CD82, a tumor metastasis suppressor. *Cancer Letters*.

[57] Xu JH, Guo XZ, Ren LN, Shao LC, Liu MP (2008). KAI1 is a potential target for anti-metastasis in pancreatic cancer cells. *World Journal of Gastroenterology*.

[58] Wu C-Y, Yan J, Yang Y-F (2011). Overexpression of KAI1 induces autophagy and increases MiaPaCa-2 cell survival through the phosphorylation of extracellular signal-regulated kinases. *Biochemical and Biophysical Research Communications*.

[59] Maher JC, Krishan A, Lampidis TJ (2004). Greater cell cycle inhibition and cytotoxicity induced by 2-deoxy-D-glucose in tumor cells treated under hypoxic vs aerobic conditions. *Cancer Chemotherapy and Pharmacology*.

[60] Xi H, Kurtoglu M, Liu H (2011). 2-Deoxy-d-glucose activates autophagy via endoplasmic reticulum stress rather than ATP depletion. *Cancer Chemotherapy and Pharmacology*.

[61] Yang S, Kimmelman AC (2011). A critical role for autophagy in pancreatic cancer. *Autophagy*.

[62] Udelnow A, Kreyes A, Ellinger S (2011). Omeprazole inhibits proliferation and modulates autophagy in pancreatic cancer cells. *PLoS ONE*.

[63] de Milito A, Fais S (2005). Proton pump inhibitors may reduce tumour resistance. *Expert Opinion on Pharmacotherapy*.

[64] de Milito A, Iessi E, Logozzi M (2007). Proton pump inhibitors induce apoptosis of human B-cell tumors through a caspase-independent mechanism involving reactive oxygen species. *Cancer Research*.

[65] Guo JY, Chen H-Y, Mathew R (2011). Activated Ras requires autophagy to maintain oxidative metabolism and tumorigenesis. *Genes and Development*.

[66] Martinez-Outschoorn UE, Lin Z, Trimmer C (2011). Cancer cells metabolically "fertilize" the tumor microenvironment with hydrogen peroxide, driving the Warburg effect: implications for PET imaging of human tumors. *Cell Cycle*.

